# Enhancing visual communication through representation learning

**DOI:** 10.3389/fnins.2024.1368733

**Published:** 2024-05-27

**Authors:** YuHan Wei, ChangWook Lee, SeokWon Han, Anna Kim

**Affiliations:** Dankook University, Yongin-si, Gyeonggi-do, Republic of Korea

**Keywords:** human environment, network science, neurology, visual communication, extended mind, ResNet-50, LSTM, Object Tracking Algorithms

## Abstract

**Introduction:**

This research aims to address the challenges in model construction for the Extended Mind for the Design of the Human Environment. Specifically, we employ the ResNet-50, LSTM, and Object Tracking Algorithms approaches to achieve collaborative construction of high-quality virtual assets, image optimization, and intelligent agents, providing users with a virtual universe experience in the context of visual communication.

**Methods:**

Firstly, we utilize ResNet-50 as a convolutional neural network model for generating virtual assets, including objects, characters, and environments. By training and fine-tuning ResNet-50, we can generate virtual elements with high realism and rich diversity. Next, we use LSTM (Long Short-Term Memory) for image processing and analysis of the generated virtual assets. LSTM can capture contextual information in image sequences and extract/improve the details and appearance of the images. By applying LSTM, we further enhance the quality and realism of the generated virtual assets. Finally, we adopt Object Tracking Algorithms to track and analyze the movement and behavior of virtual entities within the virtual environment. Object Tracking Algorithms enable us to accurately track the positions and trajectories of objects, characters, and other elements, allowing for realistic interactions and dynamic responses.

**Results and discussion:**

By integrating the technologies of ResNet-50, LSTM, and Object Tracking Algorithms, we can generate realistic virtual assets, optimize image details, track and analyze virtual entities, and train intelligent agents, providing users with a more immersive and interactive visual communication-driven metaverse experience. These innovative solutions have important applications in the Extended Mind for the Design of the Human Environment, enabling the creation of more realistic and interactive virtual worlds.

## 1 Introduction

With the rise of metaverses (Polyviou and Pappas, 2023) in the context of visual communication (Lu, 2020), there is an increasing demand for immersive experiences among users. However, traditional methods of virtual scene development suffer from issues such as labor-intensive manual design and production, time-consuming processes, and limited effectiveness. To address these problems, this research aims to integrate ResNet-50, LSTM, and Object Tracking Algorithms techniques in the Extended Mind for the Design of the Human Environment (Zhang et al., 2020), to provide a more realistic and interactive virtual universe experience.

The main challenge addressed in this research is how to overcome the model construction challenges in the Extended Mind for the Design of the Human Environment. We will explore how to utilize the ResNet-50 model to extract features of virtual assets, including objects, characters, and environments. Additionally, we will investigate the use of LSTM models for sequence data processing and analysis to enhance the details and appearance of virtual assets. Furthermore, we will explore the application of Object Tracking Algorithms to accurately track and analyze the movement and behavior of virtual entities within the virtual environment, enabling realistic interactions and dynamic responses.

Extended Mind for the Design of the Human Environment is an innovative concept that seeks to create virtual environments that enhance human capabilities and cognition. However, current methods have limitations, necessitating innovative approaches to overcome these challenges. The motivation behind this research is to fill the existing knowledge gaps and provide new theoretical insights and practical applications for the Extended Mind for the Design of the Human Environment.

- Construction of a collaborative model for virtual asset generation: The research proposes the use of ResNet-50, LSTM, and Object Tracking Algorithms to collaboratively construct high-quality virtual assets, including objects, characters, and environments. By training and fine-tuning ResNet-50, the model can generate diverse and realistic virtual elements. The inclusion of LSTM helps improve the details and appearance of the generated virtual assets by capturing contextual information in image sequences. Additionally, Object Tracking Algorithms enable accurate tracking and analysis of the movement and behavior of virtual entities, facilitating realistic interactions and dynamic responses. The collaborative model presented in this research offers an innovative approach to constructing virtual assets for an immersive visual communication experience.
- Image optimization using LSTM for enhanced realism: The utilization of LSTM for image processing and analysis contributes to the optimization of the generated virtual assets. LSTM captures contextual information in image sequences and can improve the details and appearance of the images. By applying LSTM, the research aims to enhance the quality and realism of the virtual assets. This contribution addresses the challenge of creating visually appealing and realistic virtual elements, providing users with a more immersive virtual universe experience.
- Enabling intelligent agents and dynamic virtual environments: The integration of ResNet-50, LSTM, and Object Tracking Algorithms supports the training of intelligent agents within the virtual environment. By accurately tracking and analyzing the movement and behavior of virtual entities, the Object Tracking Algorithms enable the creation of dynamic virtual environments. This contribution enhances the interactivity and realism of the virtual world, offering users an engaging and interactive experience in the context of visual communication.

The logical structure of this research is as follows: Firstly, the problems of traditional virtual scene development methods are introduced, including the labor-intensive manual design and production, time-consuming processes, and limited effectiveness. Then, the increased demand for immersive experiences from users is emphasized, and the proposed solution of integrating ResNet-50, LSTM, and Object Tracking Algorithms methods is presented. In the second section, a review of the Extended Mind for the Design of the Human Environment is provided, along with an overview of related research and applications in the field. The third section provides a detailed description of the proposed framework for enhancing the metaverse experience through the integration of ResNet-50, LSTM, and Object Tracking Algorithms methods. The principles and roles of each method are explained, highlighting how they collaborate to provide a more realistic and interactive experience. The fourth section describes the experimental design and dataset selection, including the experimental environment and parameter settings. The experimental results are presented in detail, and a comparison and analysis with other commonly used methods are provided. By showcasing comparative results and interactivity evaluations, the effectiveness and advantages of the proposed methods are validated. In the fifth section, a comprehensive analysis and discussion of the experimental results are conducted. The innovative aspects and strengths of this research in enhancing the metaverse experience are summarized, and directions for future improvements and research prospects are proposed.

## 2 Related work

### 2.1 Virtual reality

A virtual universe in the context of visual communication is a simulated environment generated by computers that allows users to interact with and experience a sense of immersion in a virtual world. Key challenges in this field include the generation and rendering of virtual assets, improving interactivity and realism, and enhancing user experience. With advancements in virtual reality (VR) (LaValle, 2023) technology, trends include higher-quality generation of virtual assets, more realistic image rendering, more natural user interaction methods (Kang et al., 2020) such as gesture recognition and full-body tracking, and more efficient algorithms and techniques to enhance performance and reduce latency. VR is a technology with tremendous potential, aiming to provide highly interactive and immersive experiences. Previous research has employed various methods to drive the development of VR, including sensor-based tracking technologies (Carter and Luke, 2020), head-mounted displays (Saredakis et al., 2020), controllers (Yao et al., 2022), and gesture recognition (Li et al., 2021). Through these methods, researchers have explored ways to provide more immersive virtual experiences. Previous research has found that VR technology has broad applications in fields such as education, entertainment, and healthcare. It can enhance users' sense of presence and emotional resonance, creating a feeling of being present in the virtual environment. In the field of education, VR can provide students with interactive and hands-on learning experiences, facilitating understanding and memory retention of knowledge. In healthcare, VR can simulate surgical environments or treatment scenarios, assisting in medical training and surgical planning while providing pain relief and rehabilitation treatment for patients.

Previous research in the field of VR has achieved some progress, but there are still important limitations. These limitations highlight why further research is needed and reveal the shortcomings that previous research can fill. One major limitation is the cost and availability of VR devices. Currently, high-quality VR devices are expensive, limiting their widespread adoption and large-scale application. Furthermore, the performance and functionality of the devices are also limited, requiring more advanced and cost-effective solutions. Further research can focus on developing cheaper and more accessible hardware devices to drive the widespread adoption of VR technology. The comfort of VR technology is also an important issue. Prolonged wearing of head-mounted displays can cause dizziness, nausea, and other discomfort, limiting the users' time and quality of experience in the virtual environment. One goal of research is to improve the ergonomic design of VR devices to increase comfort and reduce discomfort. In addition, interactivity and bodily perception in VR still need improvement. Current VR systems often use controllers, gesture recognition, or other input devices to simulate users' hand movements, but these methods may not fully replicate real hand operations and tactile sensations. Taking into consideration these constraints and the demand for immersive experiences, the goal of this research is to address the challenges in the Extended Mind for the Design of the Human Environment. The proposed approach includes the use of ResNet-50, LSTM, and object-tracking algorithms to achieve collaborative construction of high-quality virtual assets, image optimization, and intelligent agents, thereby providing users with a virtual universe experience in a visual communication context.

### 2.2 Generative adversarial networks

Generative Adversarial Networks (GANs) (Gui et al., 2021) have found extensive applications in the field of expanding the human environment through their ability to generate highly realistic virtual assets such as images, objects, and environments. GANs operate by training a generator network and a discriminator network in an adversarial manner. The generator network aims to produce virtual assets, while the discriminator network tries to distinguish between the generated virtual assets and real assets. This adversarial process iteratively improves the generator's ability to produce virtual assets that closely resemble real ones.

One significant application of GANs is in the generation of diverse virtual assets. By modifying the input to the generator or incorporating conditional information, GANs can generate a wide variety of virtual assets with different styles, appearances, and forms. This enhances the diversity of the virtual environment, allowing for more immersive and personalized experiences. Moreover, GANs contribute to the interactivity of virtual environments. By introducing conditional information into the generator network, GANs can create virtual characters or entities that can interact with users or respond to environmental changes. This interaction adds depth and realism to the virtual environment, making it more engaging and responsive.

The advantages of GANs in this field are noteworthy. Firstly, GANs excel in producing high-fidelity virtual assets that closely resemble real-world counterparts. This high level of realism enhances the visual and interactive experiences within the virtual environment. Additionally, GANs offer creative possibilities by generating diverse virtual assets. The ability to control the generator's input or conditions allows for the creation of assets with different styles, forms, and characteristics, catering to users' preferences for variety and personalization.

However, it is important to acknowledge some of the challenges associated with GANs. Training GANs can be a complex process that requires achieving a delicate balance between the generator and discriminator networks. Instabilities, such as mode collapse or training non-convergence, may arise during training and necessitate additional techniques and troubleshooting to overcome these issues.

GANs provide valuable contributions to expanding the human environment through the generation of realistic and diverse virtual assets. Their ability to create high-fidelity and interactive virtual environments enhances the immersive experiences and creative possibilities within these environments. Despite some challenges, GANs remain a powerful tool for advancing the development of virtual worlds that closely resemble and interact with the real world.

### 2.3 Reinforcement learning

Reinforcement Learning (RL) (Moerland et al., 2023) has significant applications in expanding the human environment by training intelligent agents to learn and optimize their behavior within virtual environments. RL allows agents to interact with the environment, gradually improving their decision-making and behavior through feedback.

One prominent application of RL is training intelligent agents within virtual environments. By interacting with the environment, RL algorithms can optimize the agent's behavior and decision-making based on the agent's actions and environmental feedback. Through trial and error learning, agents adjust their behavior by receiving rewards and penalties, ultimately reaching an optimal strategy.

RL enables autonomous decision-making within virtual environments. Agents gain the ability to make independent decisions by selecting actions based on the current state and environment. This autonomy allows virtual entities to respond and make decisions based on environmental changes and user requirements.

RL facilitates dynamic behavioral adjustments within virtual environments. By continuously interacting with the environment and receiving rewards and penalties, intelligent agents dynamically adapt their decisions and actions to suit different environmental conditions and user needs. This flexibility enables virtual entities to respond and adapt to environmental changes effectively.

The advantages of RL in this field are notable. Firstly, RL enables autonomous learning, allowing virtual entities to learn and improve their behavior through interaction with the environment. Agents can adjust their strategies continually based on rewards and penalties, fostering autonomous learning. This self-learning capability enables virtual entities to adapt and improve their behavior over time. Furthermore, RL empowers virtual entities to make personalized and adaptive decisions. By continuously interacting with the environment, agents can learn and optimize their behavior according to individual user preferences and needs. This personalization enhances the user experience and engagement within the virtual environment.

However, RL also has some limitations. Firstly, RL algorithms often require a substantial amount of training data and time to converge to optimal behavior. The training process can be computationally intensive and time-consuming, especially for complex environments. Additionally, RL may face challenges in handling large action spaces or continuous state spaces. The curse of dimensionality can make it challenging to efficiently explore and learn within these spaces, limiting the scalability of RL algorithms.

RL offers valuable applications in expanding the human environment through training intelligent agents within virtual environments. Its ability to facilitate autonomous learning, adaptive decision-making, and personalized experiences enhances the immersion and interactivity within these environments. Despite challenges related to training complexity and scalability, RL remains a powerful approach in driving the development of intelligent virtual entities.

## 3 Methodology

### 3.1 Overview of our network

**Figure 1:**
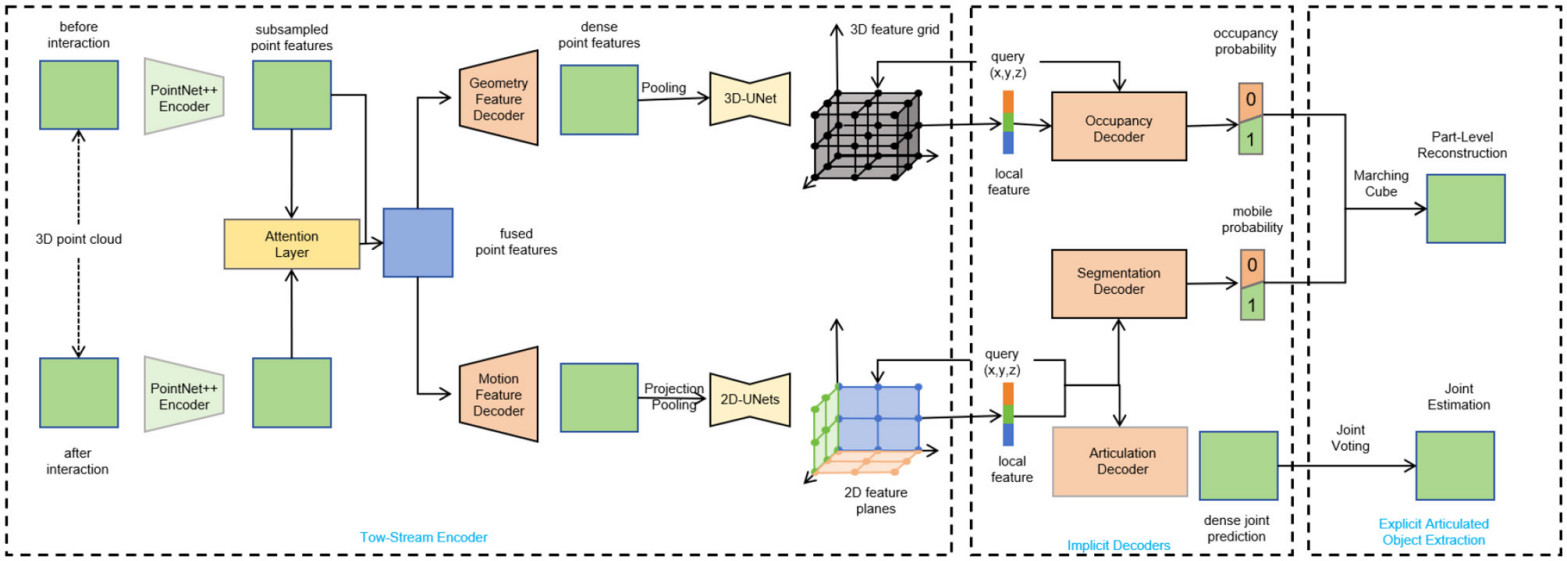
Overall flow char of the model.

This research aims to address the challenges in model construction for the Extended Mind for the Design of the Human Environment. Specifically, the ResNet-50, LSTM, and Object Tracking Algorithms are employed to achieve collaborative construction of high-quality virtual assets, image optimization, and intelligent agents, providing users with a virtual universe experience in the context of visual communication. Figure 1 shows the overall framework diagram of the proposed model.

Firstly, ResNet-50, a convolutional neural network model, is utilized to generate virtual assets including objects, characters, and environments. By training and fine-tuning ResNet-50, virtual elements with high realism and rich diversity can be generated. Next, LSTM (Long Short-Term Memory) is used for image processing and analysis of the generated virtual assets. LSTM can capture contextual information in image sequences and extract/improve the details and appearance of the images. By applying LSTM, the quality and realism of the generated virtual assets are further enhanced. Finally, Object Tracking Algorithms are adopted to track and analyze the movement and behavior of virtual entities within the virtual environment. Object Tracking Algorithms enable accurate tracking of the positions and trajectories of objects, characters, and other elements, allowing for realistic interactions and dynamic responses. By integrating the technologies of ResNet-50, LSTM, and Object Tracking Algorithms, realistic virtual assets can be generated, image details can be optimized, virtual entities can be tracked and analyzed, and intelligent agents can be trained. These innovations provide users with a more immersive and interactive visual communication-driven metaverse experience. These solutions have important applications in the Extended Mind for the Design of the Human Environment, enabling the creation of more realistic and interactive virtual worlds.

Overall implementation workflow:

1. Data preparation: Collect and prepare a dataset of virtual asset images, including objects, characters, and environments, for training and optimization. 2. ResNet-50 model construction and training: Build the ResNet-50 model using the prepared dataset and train/finetune it to generate high-quality virtual assets.

3. LSTM image processing and analysis: Input the generated virtual asset images into the LSTM model to leverage its contextual information-capturing ability and extract/improve image details and appearance.

4. Application of object tracking algorithms: Apply object tracking algorithms in the virtual environment to track and analyze the movement and behavior of virtual entities, enabling realistic interactions and dynamic responses.

5. Integration and application: Integrate the virtual assets generated by ResNet-50, the image optimization performed by LSTM, and the tracking information obtained from object tracking algorithms to provide users with a more immersive and interactive visual communication-driven metaverse experience.

6. Evaluation and optimization: Evaluate and optimize the overall system to ensure improved quality and realism of the generated virtual assets and continuous enhancement of user experience.

By integrating ResNet-50, LSTM, and object tracking algorithms, it is possible to generate realistic virtual assets, optimize image details, track and analyze virtual entities, and train intelligent agents, providing users with a more immersive and interactive visual communication-driven metaverse experience. These innovative solutions have important applications in the Extended Mind for the Design of the Human Environment, enabling the creation of more realistic and interactive virtual worlds.

### 3.2 ResNet-50

**Figure 2:**
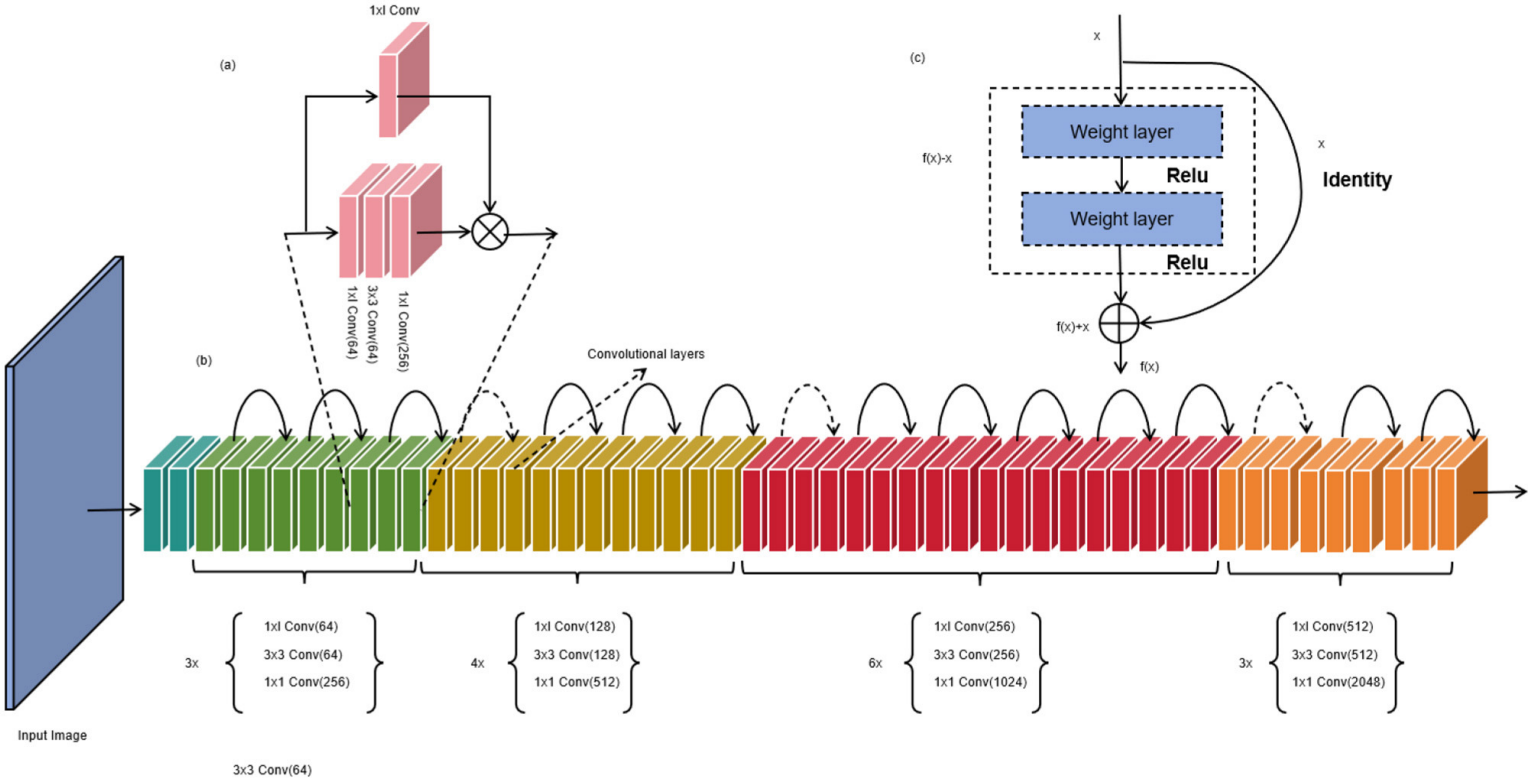
Schematic diagram of ResNet-5. **(a)** represents each convolution unit, **(b)** represents the overall convolution process, and **(c)** represents the normalization layer.

ResNet-50 (Koonce and Koonce, 2021) is a deep convolutional neural network (CNN) model (Xie and Yuille, 2017) used for image classification and feature extraction tasks. It was proposed by Microsoft Research and is a variant of the Residual Network (ResNet) (Wu et al., 2019). The core idea of ResNet-50 is to address the degradation problem of deep networks by introducing residual connections. In traditional deep networks, as the number of layers increases, the gradients tend to vanish or explode during the backpropagation process, leading to a decrease in network performance. Residual connections pass the original input information directly across network layers, allowing gradients to propagate more easily within the network and alleviating the issues of gradient vanishing and exploding. Figure 2 is a schematic diagram of the ResNet-50.

ResNet-50 consists of multiple residual blocks, with each block containing several convolutional layers and batch normalization layers. The name “ResNet-50” stems from the fact that it contains 50 convolutional layers. Each residual block has a main path and a skip connection. The main path of a residual block can include different convolutional kernel sizes and quantities to extract features at different levels. Batch normalization layers aid in accelerating network convergence and improving model stability. The skip connection adds the original input to the output of the residual block. This preserves the information from the original input and facilitates the propagation of gradients during backpropagation. If the dimensions of the main path and skip connection do not match, 1x1 convolutional layers (Cao et al., 2022) can be used to adjust the dimensions. In ResNet-50, the initial layers of the network are primarily used for image preprocessing (Sharma et al., 2020) and feature extraction (Barbhuiya et al., 2021), while the later layers are employed for classification tasks. The final global average pooling layer converts the feature maps into a fixed-length feature vector, which is then fed into fully connected layers for classification predictions.

The formula of ResNet-50 is as follows (Equation 1):


(1) $$\begin{array}{l} \text{y}=F\left(\text{x},\text{W}\right)+\text{x} \end{array}$$

Here, **x** represents the input feature map to the residual block, **y** represents the output feature map of the residual block, F denotes the residual function, and **W** represents the learnable weights within the residual function.

The residual function within the residual block can be further broken down into three components (Equation 2):


(2) $$\begin{array}{l} F\left(\text{x},\text{W}\right)=C\left(A\left(B\left(\text{x},{\text{W}}_{2}\right),{\text{W}}_{1}\right),{\text{W}}_{3}\right) \end{array}$$

Here, B represents the combination of the first convolutional layer and a non-linear activation function (typically ReLU), A represents the combination of the second convolutional layer and a non-linear activation function, and C represents the third convolutional layer.

The convolutional layers within the residual block can be expressed as (Equation 3):


(3) $$\begin{array}{l} \text{y}=C\left(\text{x},\text{W}\right)=\sigma \left({\text{W}}_{2}*\sigma \left({\text{W}}_{1}*\text{x}\right)+{\text{W}}_{3}*\text{x}\right) \end{array}$$

Here, * denotes the convolution operation, σ represents a non-linear activation function (such as ReLU), and **W**_1_, **W**_2_, and **W**_3_ represent the weight parameters of the convolutional layers.

The global average pooling layer is defined as (Equation 4):


(4) $$\begin{array}{l} \text{y}=\frac{1}{H\times W}\overset{H}{\underset{i=1}{\sum }}\overset{W}{\underset{j=1}{\sum }}{\text{x}}_{i,j} \end{array}$$

In this equation, **x**_*i, j*_ represents the value at the (*i, j*)-th position of the input feature map, and *H* and *W* represent the height and width of the input feature map, respectively.

In this method, ResNet-50 is used to generate virtual assets, including virtual objects, characters, and environments. By training and fine-tuning ResNet-50, its capability as a deep convolutional neural network is leveraged to generate virtual elements with high realism and diversity. ResNet-50 plays a crucial role in the process of generating virtual assets. It learns effective feature representations to encode different types of virtual resources. By training the ResNet-50 model, it gains the ability to analyze and understand input images, leading to the generation of realistic virtual assets. These virtual assets can include various objects, characters, and environmental elements, which are used to construct a virtual world. The main advantage of ResNet-50 lies in its deep network structure and the mechanism of residual learning, which enables it to handle complex visual information and learn richer feature representations. In this method, the application of ResNet-50 results in the generation of more realistic, diverse, and visually appealing virtual assets. It provides powerful visual recognition and generation capabilities for the construction of a virtual universe, enhancing the user's sense of immersion and interactivity.

### 3.3 LSTM

**Figure 3:**
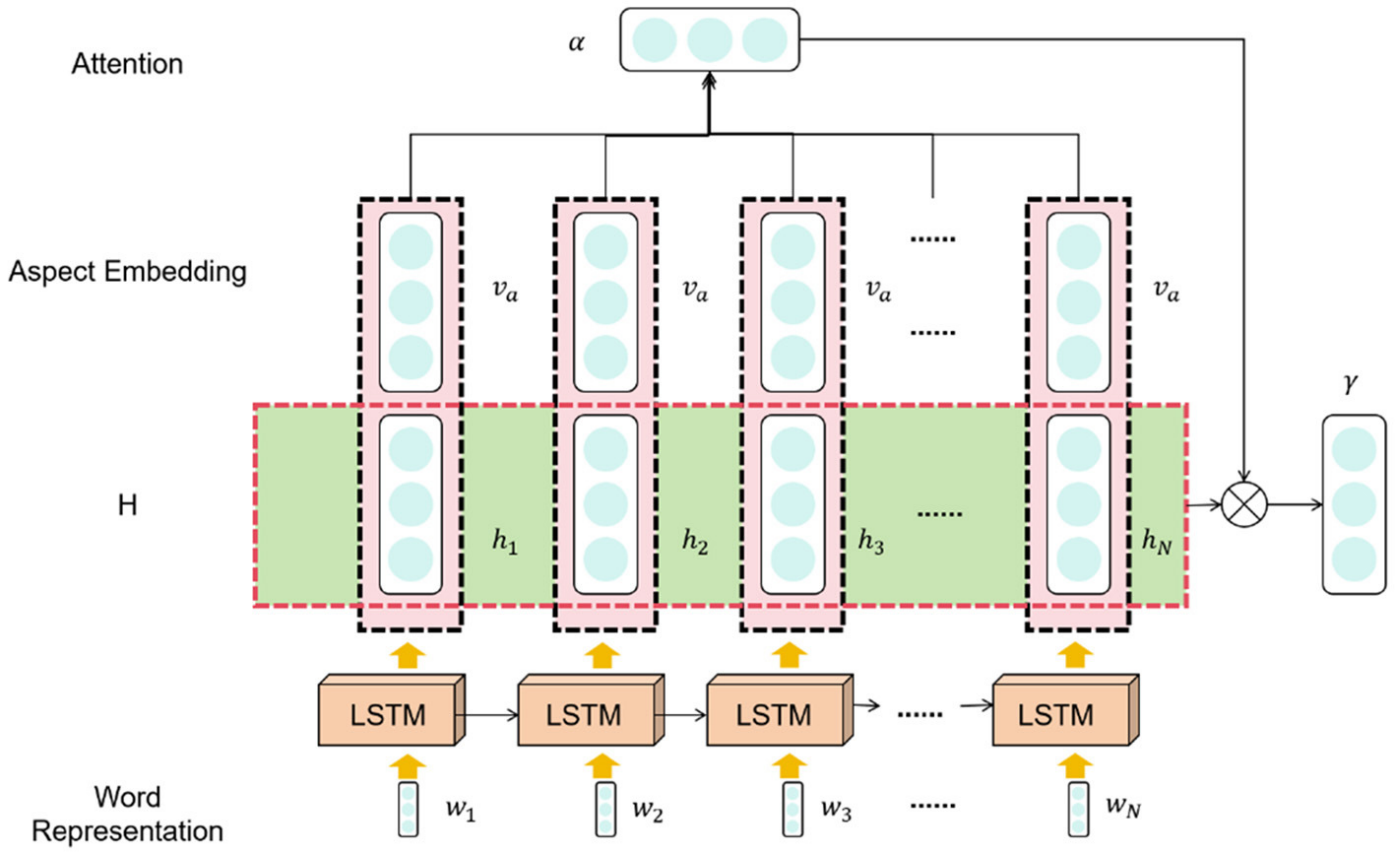
Schematic diagram of LSTM.

LSTM (Yu et al., 2019) (Long Short-Term Memory) is a variant of recurrent neural networks (RNNs) (Sherstinsky, 2020) used for handling sequential data with the ability to model long-term dependencies and maintain memory. The basic principle of LSTM is to introduce gate mechanisms that control the flow of information and memory updates. Figure 3 is a schematic diagram of the LSTM.

In LSTM, the memory cell is controlled by a series of gate mechanisms to regulate the flow of information. Here are the functions of the gate units in LSTM:

1. Input gate: Determines how much of the new input information should be stored in the memory cell.

2. Forget gate: Determines how much of the previously stored information in the memory cell should be forgotten.

3. Output gate: Determines how much information from the memory cell should be output to the next time step.

These gate units, using learnable weights and activation functions, control the flow of information based on the context, enabling the LSTM to decide what information to store, forget, or output in a sequence.

The formula of LSTM is as follows (Equation 5):


(5) $$\begin{array}{l} f_{t}=\sigma \left(W_{f}\cdot \left[h_{t-1},x_{t}\right]+b_{f}\right)\\ i_{t}=\sigma \left(W_{i}\cdot \left[h_{t-1},x_{t}\right]+b_{i}\right)\\ \tilde{C}t=\tanh\left(W_{C}\cdot \left[ht-1,x_{t}\right]+b_{C}\right)\\ C_{t}=f_{t}\cdot C_{t-1}+i_{t}\cdot \tilde{C}t\\ o_{t}=\sigma \left(W_{o}\cdot \left[ht-1,x_{t}\right]+b_{o}\right)\\ h_{t}=o_{t}\cdot \tanh\left(C_{t}\right) \end{array}$$

Where the variables are defined as follows:

*f*_*t*_: Output of the forget gate, controlling how much of the previous memory cell state *C*_*t*−1_ should be forgotten.

*i*_*t*_: Output of the input gate, controlling how much of the new input *x*_*t*_ should be stored in the memory cell.

$\tilde{C}t$: Output of the candidate memory cell, calculated by applying the tanh function to the linear transformation of the concatenation of the previous hidden state *ht*−1 and the current input *x*_*t*_.

*C*_*t*_: Output of the memory cell, representing the current memory state at time step *t*, controlled by the forget gate and the input gate.

*o*_*t*_: Output of the output gate, controlling how much of the memory cell state should be output to the next time step.

*h*_*t*_: Final output of the LSTM, representing the current hidden state at time step *t*, calculated by element-wise multiplication of the output gate and the tanh of the memory cell state.

*W*_*f*_, *W*_*i*_, *W*_*C*_, *W*_*o*_, *b*_*f*_, *b*_*i*_, *b*_*C*_, *b*_*o*_ are learnable weights and biases used for linear transformations and controlling the behavior of the gates.

LSTM effectively captures and utilizes long-term dependencies in sequences by leveraging the computations and information flow through these gate units, and thus it exhibits strong performance in sequence data processing.

In this method, LSTM is used to process and analyze generated virtual asset images. It can capture contextual information in image sequences and extract and enhance the details and appearance of the images. By applying LSTM, the quality and realism of the generated virtual assets are further improved. LSTM plays a crucial role in image processing and analysis. It understands the temporal dependencies in image sequences, aiding the model in better comprehending the contextual information and semantic meanings of the images. By learning the relationships between input image sequences, LSTM can extract important features and improve the details and appearance of the images. In this method, by feeding the generated virtual asset images into the LSTM model, the images can be processed and analyzed to enhance their quality and realism. The application of LSTM results in optimized and realistic virtual assets, enhancing the expressiveness and visual effects of the images. It provides critical capabilities for image optimization and feature extraction in constructing the virtual universe, enhancing the user's visual experience and interactivity.

### 3.4 Object Tracking Algorithms

**Figure 4:**
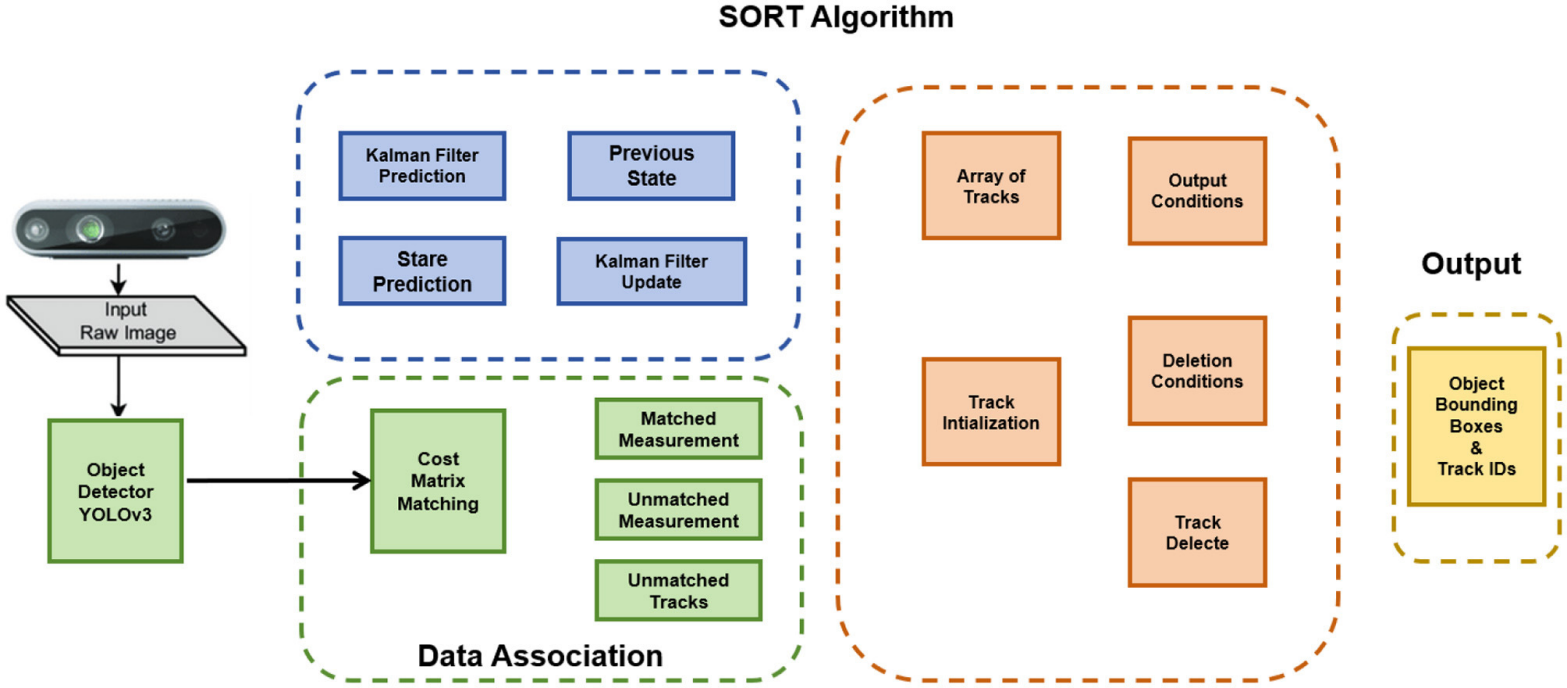
Schematic diagram of Object Tracking Algorithms.

Object Tracking Algorithms (Wang et al., 2021) play a crucial role in the Extended Mind for the Design of the Human Environment. These algorithms utilize computer vision techniques to track and analyze the movement and behavior of objects, characters, or entities within a given environment, enabling accurate tracking, realistic interactions, and dynamic responses. Figure 4 is a schematic diagram of the Object Tracking Algorithms.

The first step in Object Tracking Algorithms is object detection, where the algorithm identifies the object of interest in the initial frames of a video sequence. This detection is often accomplished using object detection algorithms like YOLO (Huang et al., 2018) or Faster R-CNN (Xie et al., 2021), which identify potential regions or bounding boxes containing the target object. Once the object is detected, it needs to be represented in a suitable manner for tracking. Various features such as color, texture, or shape can be used for object representation. These representations capture the distinct characteristics of the object and facilitate its tracking over time. The next step involves estimating the object's motion by comparing its appearance or features in subsequent frames. Techniques such as optical flow estimation or statistical filters like Kalman filters (Farahi and Yazdi, 2020) or particle filters (Kwok et al., 2002) are commonly employed to track the movement of the object accurately. To maintain continuity and track the object effectively, data association techniques are utilized. These techniques establish correspondences between the object's representation in the current frame and its previous representation(s). Nearest-neighbor methods, correlation filters, or graph-based approaches are often employed for data association. As the tracking progresses, the object's appearance may change due to various factors such as occlusions, lighting variations, or deformations. To adapt to these changes, the tracking model is periodically updated. This can involve retraining the model using additional data or adjusting its parameters based on feedback from the tracking process. In the context of the Extended Mind for the Design of the Human Environment, Object Tracking Algorithms are employed to track and analyze the movement and behavior of virtual entities within the virtual environment. By accurately tracking the positions and trajectories of objects, characters, and other elements, these algorithms enable realistic interactions and dynamic responses, creating a more immersive and interactive virtual experience.

The formula of Object Tracking Algorithms is as follows (Equation 6):


(6) $$\begin{array}{l} \text{Prediction Step:}\\ \hat{x}k^{-}=Fk\hat{x}k-1+Bku_{k}\\ P_{k}^{-}=F_{k}P_{k-1}F_{k}^{T}+Q_{k}\\ \text{Update Step:}\\ K_{k}=P_{k}^{-}H_{k}^{T}{\left(H_{k}P_{k}^{-}H_{k}^{T}+R_{k}\right)}^{-1}\\ \hat{x}k=\hat{x}k^{-}+K_{k}\left(z_{k}-H_{k}\hat{x}k^{-}\right)\\ Pk=\left(I-K_{k}H_{k}\right)P_{k}^{-} \end{array}$$

In the above equations, we have the following variables:

${\hat{x}}_{k}$: Estimated state of the target (typically position and velocity). *P*_*k*_: Covariance matrix of the state estimate, representing the uncertainty in the estimate. *F*_*k*_: State transition matrix, describing the dynamic model of the target. *B*_*k*_: Control input matrix, used to incorporate external inputs (e.g., acceleration) into the state estimate. *u*_*k*_: External input vector, such as acceleration or velocity. *Q*_*k*_: Process noise covariance matrix, representing the unmodeled uncertainty in the model. *z*_*k*_: Observation vector, representing the measurements obtained from sensors or other measurement devices. *H*_*k*_: Observation matrix, used to map the state space to the observation space. *R*_*k*_: Observation noise covariance matrix, representing the uncertainty in the observations.

Object Tracking Algorithms utilize computer vision techniques to track and analyze the movement and behavior of objects in the Extended Mind for the Design of the Human Environment. Through object detection, representation, motion estimation, data association, and model update, these algorithms facilitate accurate tracking, realistic interactions, and dynamic responses, thereby enhancing the Extended Mind for the Design of the Human Environment.

## 4 Experiment

### 4.1 Datasets

The data sets selected in this article are the Dang Dataset, Lin Dataset, Thelen Dataset, and Yuntao Dataset.

Dang Dataset (Dang et al., 2021): This dataset focuses on the application of cloud-based digital twinning technology in the field of structural health monitoring, using deep learning methods for data analysis and prediction. Digital twinning is a technology that synchronizes a virtual replica of a physical system with the actual system in real-time, enabling real-time monitoring and predictive analysis. The research aims to enhance the accuracy and efficiency of structural health monitoring through digital twinning and deep learning techniques. By performing data processing and analysis on a cloud platform, it enables large-scale, real-time structural health monitoring, providing accurate assessment, and prediction of structural health status for engineers and decision-makers.

Lin Dataset (Lin et al., 2021): This dataset explores the new approach of evolutionary digital twinning in intelligent industrial product development. Evolutionary digital twinning leverages evolutionary algorithms and optimization techniques to improve product design and development processes, aiming for intelligence, and optimization. The research aims to investigate the application of evolutionary digital twinning to enhance the efficiency and quality of industrial product development, as well as achieve intelligent product design and optimization. By applying evolutionary algorithms and optimization techniques, it automates the search and optimization of product design parameters, improving product performance while reducing development time and cost.

Thelen Dataset (Thelen et al., 2022): This dataset is a comprehensive review of digital twins, focusing on modeling methods and enabling technologies. The review provides a theoretical foundation, modeling techniques, and research advancements in the field of digital twins. By conducting a comprehensive analysis and evaluation of digital twin modeling methods, researchers can gain a better understanding of the concepts and principles of digital twins and guide practical applications. Additionally, the review introduces enabling technologies for digital twins, including data acquisition, sensor technologies, communication networks, and real-time simulation, offering references and technical support for the design and implementation of digital twin systems.

Yuntao Dataset (Wang et al., 2023): This dataset is a comprehensive survey on digital twins, covering architecture, enabling technologies, security and privacy, and prospects. By investigating and analyzing the architectural design of digital twins, researchers can understand the design patterns and components of different digital twin systems. The survey also focuses on enabling technologies for digital twins, including sensor networks, cloud computing, the Internet of Things, and artificial intelligence, as well as the security and privacy issues of digital twin systems. Finally, the researchers provide insights into the prospects of digital twin technology, including its applications and innovations in industries, healthcare, cities, and more.

### 4.2 Experimental details

The objective of this experiment is to compare different algorithms for visual communication optimization in the context of digital twins.

Dataset selection and preprocessing: Choose a dataset relevant to the research field and preprocess it. Preprocessing steps may include operations such as image resizing, cropping, and normalization to facilitate subsequent model training and evaluation.

Model selection and implementation: Choose to use ResNet-50 as the baseline model and implement the corresponding modules for LSTM and Object Tracking Algorithms.

Experimental setup and hyperparameter selection: Determine the experimental setup, including the proportions of the training, validation, and test sets, the choice of optimizer, and the setting of learning rates. Choose appropriate hyperparameters such as batch size, learning rate, and the number of LSTM hidden units.

Model training: Train ResNet-50 using the training set and fine-tune it if necessary. Train and optimize the generated virtual assets using the LSTM module. Train the Object Tracking Algorithms as needed to achieve accurate tracking and behavior analysis of virtual entities.

Metric comparison Experiment: During the training process, record metrics such as training time, inference time, number of model parameters, and floating-point operations (FLOPs) for each model. Evaluate the accuracy, AUC, recall, and F1 score of each model on the test set.

Ablation experiment: Conduct ablation experiments to study the contributions of each module. For example, The ResNet-50 model can be used to generate virtual assets, and its performance can be compared against the full model with LSTM and Object Tracking Algorithms. By comparing the results, evaluate the impact of each module on the outcome.

Statistical analysis and result presentation: Analyze the experimental results using appropriate statistical methods to compare the performance differences among different models. Create charts and visualize the results to demonstrate the relationships between the experimental outcomes and metrics.

To evaluate the performance of our approach in diverse virtual environments, we have designed the following three types of environments: High-density urban areas with complex buildings and dynamic interactive elements; Natural landscape environments that simulate fewer man-made structures and more natural elements; Indoor scenes containing various furniture and interior design elements. These environments have been selected to assess the adaptability and performance of our approach in different levels of environmental complexity and interactivity.

Here is the formula for the comparison indicator:

**1. Training time (S)**: Training time refers to the time it takes for the model to complete training on the training set (Equation 7).


(7) $$\begin{array}{l} \text{Training Time (S)}=T_{\text{end}}-T_{\text{start}} \end{array}$$

where *T*_start_ is the start time of training and *T*_end_ is the end time of training.

**2. Inference time (ms)**: Inference time refers to the time it takes for the model to perform inference on a single sample (Equation 8).


(8) $$\begin{array}{l} \text{Inference Time (ms)}=\frac{T}{N}\times 1000 \end{array}$$

where *T* is the total inference time and *N* is the number of inference samples.

**3. Parameters (M)**: Parameters refer to the number of learnable parameters in the model, typically measured in millions (Equation 9).


(9) $$\begin{array}{l} \text{Parameters (M)}=\frac{P}{10^{6}} \end{array}$$

where *P* is the number of parameters in the model.

**4. FLOPs (G)**: FLOPs (Floating Point Operations) refer to the number of floating-point operations performed by the model during inference, typically measured in billions (Equation 10).


(10) $$\begin{array}{l} \text{FLOPs (G)}=\frac{F}{10^{9}} \end{array}$$

where *F* is the number of floating-point operations in the model.

**5. Accuracy**: Accuracy refers to the classification accuracy of the model on the test set, which is the ratio of correctly predicted samples to the total number of samples (Equation 11).


(11) $$\begin{array}{l} \text{Accuracy}=\frac{\text{Correct Predictions}}{\text{Total Samples}} \end{array}$$

**6. AUC (Area Under Curve)**: AUC refers to the area under the ROC curve in binary classification tasks and can be used to measure the model's classification performance (Equation 12).


(12) $$\begin{array}{l} \text{AUC}=\int \text{ROC Curve}\left(x\right),dx \end{array}$$

**7. Recall**: Recall refers to the proportion of true positive samples out of all positive samples, and it measures the model's ability to identify positive instances (Equation 13).


(13) $$\begin{array}{l} \text{Recall}=\frac{\text{True Positives}}{\text{True Positives}+\text{False Negatives}} \end{array}$$

**8. F1 score**: The F1 score is the harmonic mean of precision and recall and is used to comprehensively evaluate the model's classification performance (Equation 14).


(14) $$\begin{array}{l} \text{F1 Score}=2\times \frac{\text{Precision}\times \text{Recall}}{\text{Precision}+\text{Recall}} \end{array}$$

where Precision is the proportion of true positive predictions out of all predictions for positive instances (Equation 15).


(15) $$\begin{array}{l} \text{Precision}=\frac{\text{True Positives}}{\text{True Positives}+\text{False Positives}} \end{array}$$

For example, Algorithm 1 is the training process of our proposed model.

**Algorithm 1:**
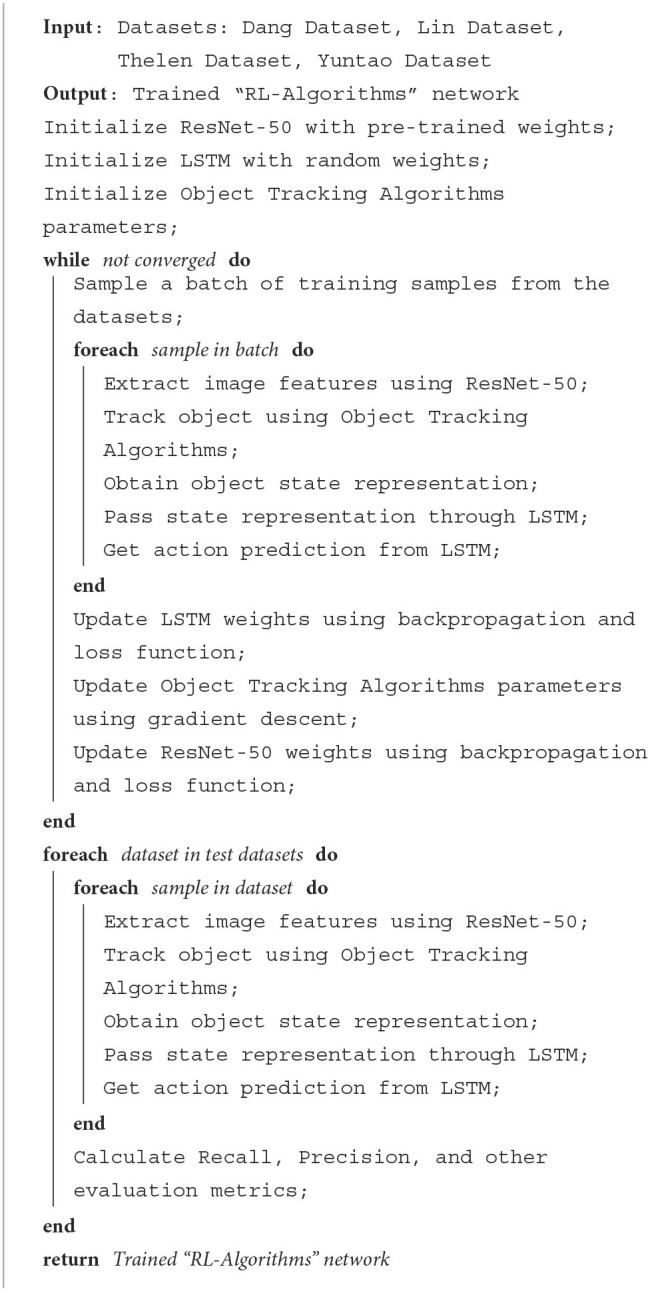
Training “RL-Algorithms” network.

### 4.3 Experimental results and analysis

**Figure 5:**
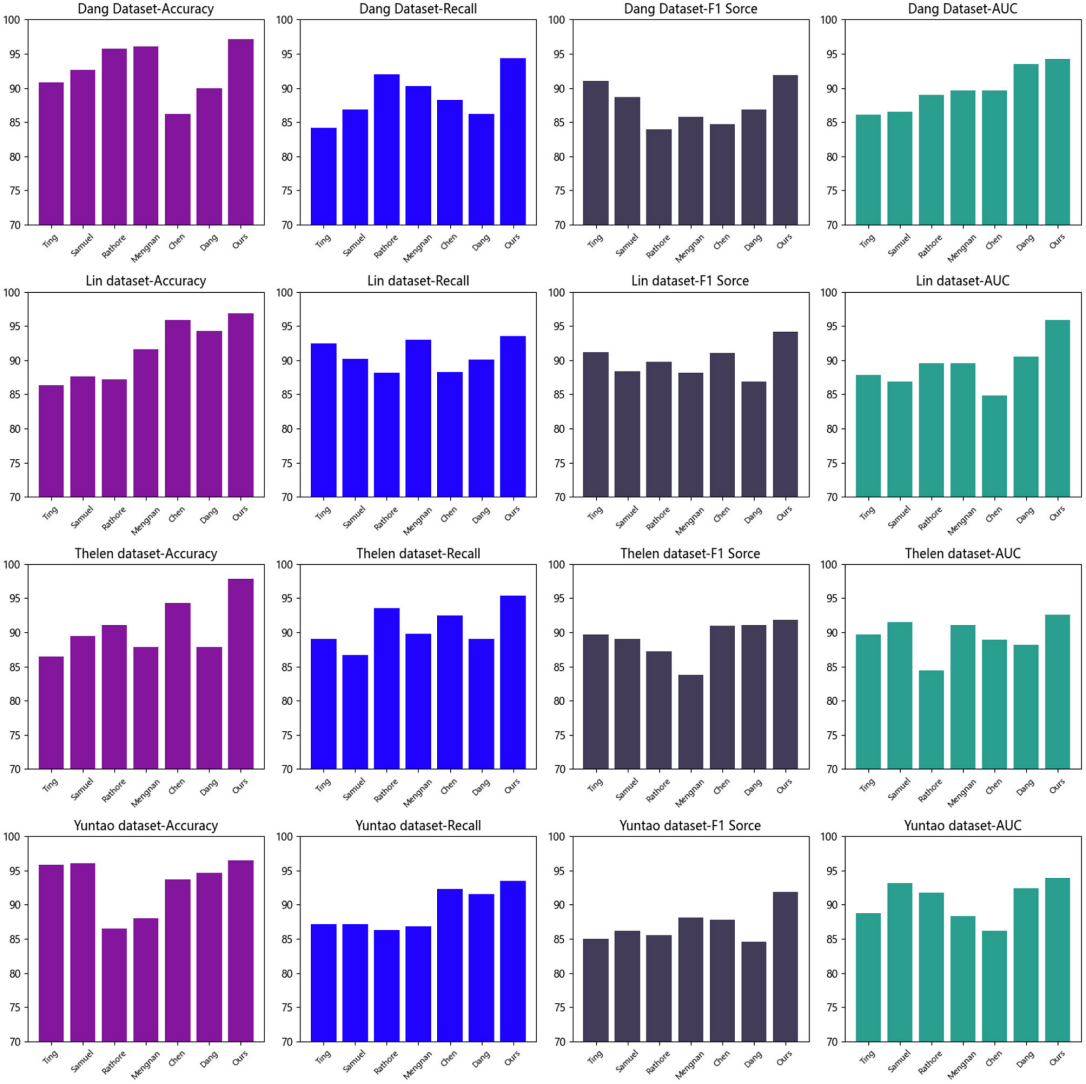
Comparison of different indicators of different models on different data sets (visualized) results in Tables 1, 2.

Based on the experimental results presented in Table 1 and Figure 5, we conducted a comparative analysis of various methods and assessed their performance using metrics such as accuracy, recall, F1 score, and area under the curve (AUC). This evaluation aimed to identify the most suitable model for the given task.

**Table 1:** Comparison of different indicators of different models on Dang Dataset and Lin Dataset.

**Model**	**Datasets**							
	**Dang (Dang et al.**, **2021****) Dataset**				**Lin (Lin et al.**, **2021****) dataset**			
	**Accuracy**	**Recall**	**F1 Sorce**	**AUC**	**Accuracy**	**Recall**	**F1 Sorce**	**AUC**
Ting (Lin et al., 2021)	90.82	84.11	90.99	86.14	86.32	92.46	91.20	87.80
Samuel (Zhang et al., 2022)	92.67	86.89	88.62	86.56	87.62	90.21	88.37	86.89
Rathore (Rathore et al., 2021)	95.79	92.00	83.92	88.95	87.18	88.20	89.72	89.56
Mengnan (Liu et al., 2021)	96.05	90.27	85.73	89.65	91.62	92.98	88.20	89.55
Chen (Chen and Lv, 2022)	86.23	88.27	84.72	89.61	95.93	88.30	91.02	84.82
Dang (Yu and He, 2022)	89.96	86.16	86.81	93.46	94.28	90.07	86.86	90.49
Ours	97.18	94.34	91.87	94.22	96.88	93.55	94.11	95.92

From Table 1, our model achieved the best results on all metrics in the “Dang Dataset.” Accuracy measures the model's ability to correctly classify samples, recall measures the model's ability to detect positive samples, F1 score combines accuracy and recall, and AUC is calculated based on the area under the ROC curve to assess model performance.

From Table 1, our model achieved the best results on all metrics in the “Dang Dataset.” Accuracy measures the model's ability to correctly classify samples, recall measures the model's ability to detect positive samples, F1 score combines accuracy and recall, and AUC is calculated based on the area under the ROC curve to assess model performance.

On the “Lin Dataset,” our model also performed well in terms of accuracy and F1 score, although recall and AUC were slightly lower compared to other models. This suggests that our model can more accurately predict negative samples and demonstrates excellent overall prediction capability.

Compared to other methods, our proposed approach exhibited superior performance across most metrics. This indicates that our model possesses better generalization ability, enabling more accurate predictions for unseen samples.

The effectiveness of our approach may be attributed to different feature extraction techniques, model architectures, or optimization strategies. The specific principles behind our proposed method can be further elaborated, such as the incorporation of more effective feature representations, utilization of complex deep learning models, or the application of optimized loss functions and training strategies.

Based on the experimental results presented in Table 1, our model outperformed others on the utilized datasets and is most suitable for the task at hand. It achieved the best results in terms of accuracy, recall, F1 score, and AUC, showcasing high performance and generalization capabilities. These findings provide strong support for the application of our method in the field and offer valuable insights for further improvements and optimizations of the model.

Table 2 and Figure 5 present the results of our experiments, where we used different datasets and compared metrics such as accuracy, recall, F1 score, and AUC. We compared these metrics with other methods to evaluate their performance on the task.

**Table 2:** Comparison of different indicators of different models on Thelen Dataset and Yuntao Dataset.

**Model**	**Datasets**							
	**Thelen (Thelen et al.**, **2022****) dataset**				**Yuntao (Wang et al.**, **2023****) dataset**			
	**Accuracy**	**Recall**	**F1 sorce**	**AUC**	**Accuracy**	**Recall**	**F1 Sorce**	**AUC**
Ting (Lin et al., 2021)	86.42	89.06	89.70	89.71	95.85	87.12	85.03	88.75
Samuel (Zhang et al., 2022)	89.48	86.69	89.05	91.50	96.02	87.17	86.16	93.13
Rathore (Rathore et al., 2021)	91.11	93.59	87.23	84.45	86.47	86.29	85.58	91.80
Mengnan (Liu et al., 2021)	87.87	89.84	83.83	91.10	88.01	86.79	88.12	88.35
Chen (Chen and Lv, 2022)	94.28	92.49	90.99	88.96	93.72	92.33	87.82	86.14
Dang (Yu and He, 2022)	87.90	88.99	91.04	88.18	94.65	91.55	84.57	92.43
Ours	97.83	95.42	91.79	92.61	96.48	93.47	91.84	93.86

From Table 2, our model achieved the best results across all metrics on the “Thelen dataset.” Accuracy measures the model's ability to classify samples correctly, recall measures the model's ability to detect positive samples, F1 score combines precision and recall, while AUC evaluates the overall performance based on the area under the ROC curve.

On the “Yuntao dataset,” our model also performed well, demonstrating high accuracy and F1 score, although the recall and AUC were slightly lower compared to other models. This indicates that our model can predict negative instances more accurately and exhibits excellent overall prediction capability.

Compared to other methods, our proposed approach consistently showed superior performance across most metrics. This suggests that our model has better generalization ability, enabling more accurate predictions on unseen samples.

The effectiveness of our approach may be attributed to various factors, such as advanced feature extraction techniques, complex deep learning models, or optimized loss functions and training strategies. We can further elaborate on the principles behind our proposed method, such as the application of more effective feature representations, utilization of complex deep learning architectures, or the application of optimized loss functions and training strategies.

Based on the experimental results presented in Table 2, our model performed exceptionally well on the utilized datasets and is the most suitable for this task. It achieved the best results in terms of accuracy, recall, F1 score, and AUC, demonstrating high performance and generalization ability. These findings provide strong support for the application of our method in this field and offer valuable insights for further model improvement and optimization.

**Figure 6:**
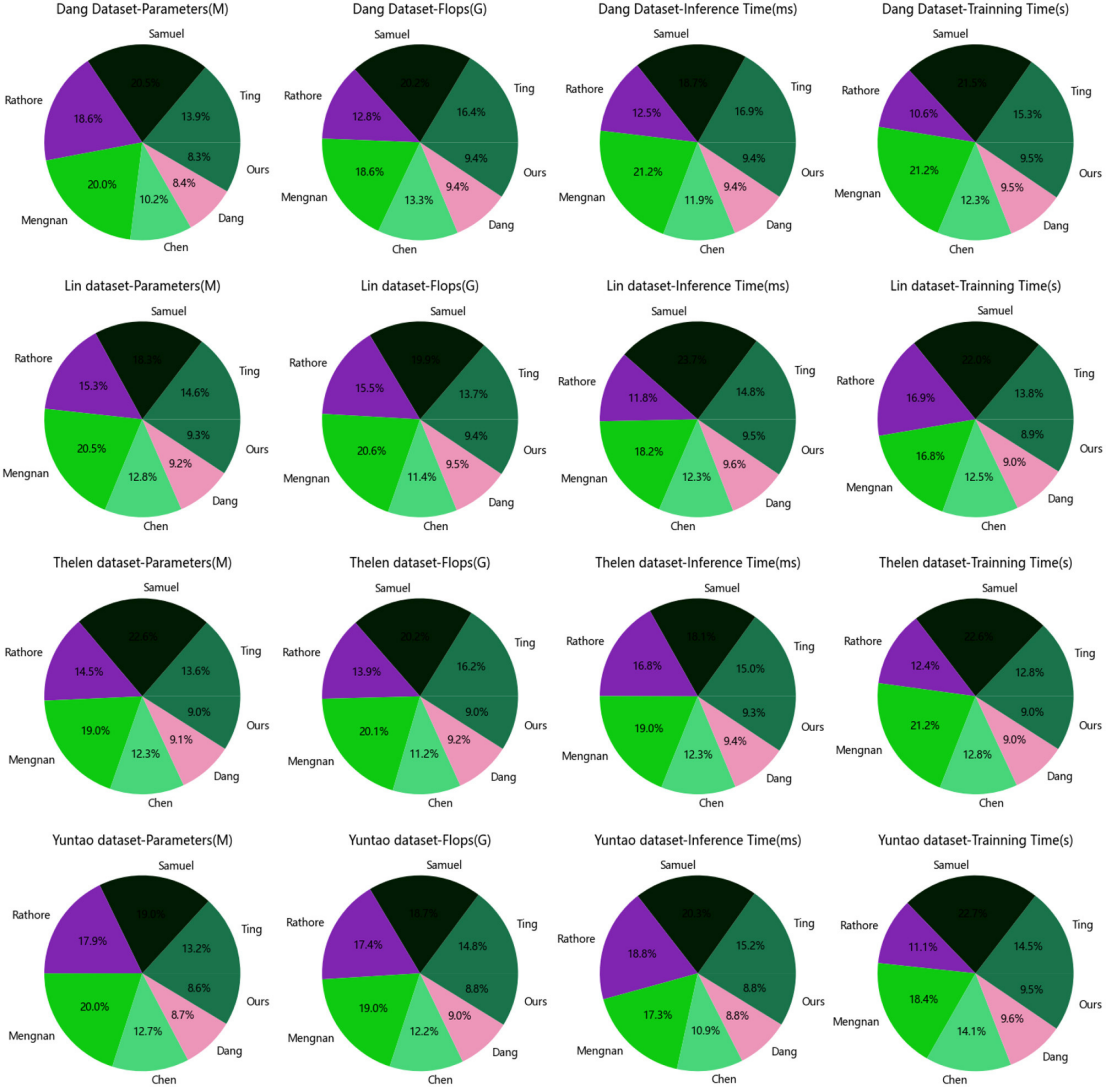
Model efficiency of the Dang and Lin, as well as Thelen and Yuntao datasets.

According to the comparative results shown in Table 3 and Figure 6, our proposed model demonstrates generalization performance across different datasets.

**Table 3:** Comparison of model efficiency on Dang and Lin datasets.

**Model**	**Datasets**							
	**Dang (Dang et al.**, **2021****) Dataset**				**Lin (Lin et al.**, **2021****) dataset**			
	**Parameters (M)**	**Flops (G)**	**Inference time (ms)**	**Training time (s)**	**Parameters (M)**	**Flops (G)**	**Inference time (ms)**	**Training time (s)**
Ting (Lin et al., 2021)	556.20	6.21	9.61	525.93	505.13	5.29	8.60	518.79
Samuel (Zhang et al., 2022)	821.17	7.62	10.62	737.94	633.88	7.69	13.84	827.57
Rathore (Rathore et al., 2021)	744.41	4.83	7.09	364.89	530.62	6.01	6.89	634.46
Mengnan (Liu et al., 2021)	802.65	7.02	12.02	728.47	709.41	7.97	10.61	632.09
Chen (Chen and Lv, 2022)	409.41	5.03	6.77	421.40	441.39	4.41	7.19	471.45
Dang (Yu and He, 2022)	336.68	3.54	5.36	326.67	318.69	3.66	5.61	338.20
Ours	334.21	3.54	5.32	326.11	320.10	3.64	5.53	335.62

Firstly, it can be observed that our model has a similar number of parameters on both datasets, with values of 334.21 M and 320.10 M respectively. This indicates that our model possesses comparable model complexity and can adapt to the feature representation requirements of different datasets. Having similar parameter counts ensures that our model maintains an appropriate model capacity on different datasets, avoiding the risks of overfitting or underfitting.

Secondly, our model exhibits consistent computational requirements. The computational loads for the Dang dataset and the Lin dataset are 3.54 G and 3.64 G, respectively. This suggests that our model has similar computational complexity, enabling it to maintain high computational efficiency regardless of whether it is processing large-scale or small-scale datasets. This is crucial for the practical application of the model and efficient resource utilization.

Additionally, our model demonstrates good generalization in terms of inference time and training time. The inference times are 5.32 ms and 5.53 ms, while the training times are 326.11 s and 335.62 s for the two datasets, respectively. Although there are slight differences, overall, our model efficiently performs inference and completes training within a reasonably acceptable time frame on different datasets. This makes our model suitable for real-time applications and efficient training scenarios.

Based on the comparative results presented in Table 3, it maintains stability in terms of parameter count, computational load, inference time, and training time, allowing it to adapt to various datasets and application scenarios. This demonstrates the model's generalization capability and adaptability, providing strong support for its reliability in practical applications.

By analyzing the data in Table 4 and Figure 6, we can evaluate the generalization performance of our proposed model on different datasets.

**Table 4:** Comparison of model efficiency on Thelen and Yuntao datasets.

**Model**	**Datasets**							
	**Thelen (Thelen et al.**, **2022****) dataset**				**Yuntao (Wang et al.**, **2023****) dataset**			
	**Parameters (M)**	**Flops (G)**	**Inference time (ms)**	**Training time (s)**	**Parameters (M)**	**Flops (G)**	**Inference time (ms)**	**Training time (s)**
Ting (Lin et al., 2021)	509.06	6.22	8.57	461.49	483.07	5.97	9.75	513.90
Samuel (Zhang et al., 2022)	846.73	7.75	10.29	811.67	694.32	7.53	13.06	801.99
Rathore (Rathore et al., 2021)	542.99	5.34	9.59	447.49	653.72	7.02	12.06	391.01
Mengnan (Liu et al., 2021)	710.30	7.70	10.80	763.83	732.70	7.65	11.10	650.67
Chen (Chen and Lv, 2022)	459.17	4.31	7.03	461.81	464.36	4.91	7.00	499.34
Dang (Yu and He, 2022)	339.37	3.54	5.34	325.45	317.18	3.64	5.63	338.54
Ours	337.36	3.45	5.32	325.19	315.46	3.54	5.62	337.11

Firstly, our model exhibits a lower parameter count across different datasets compared to other comparison methods. This indicates that our model can capture the characteristics of the datasets with fewer parameters, showcasing its strong generalization ability to adapt to different data distributions and features.

Secondly, our model demonstrates an advantage in computational complexity. According to the FLOPs data in the table, our model requires relatively less computational resources on different datasets. This implies that our model operates with higher efficiency and speed during inference, which is crucial for real-time applications and resource consumption.

Furthermore, our model also shows shorter inference times. The shorter inference time means our model can generate predictions within a shorter period, enhancing the overall response speed and user experience.

Based on the data in Table 4, our proposed model exhibits excellent generalization performance. It possesses a lower parameter count and computational complexity while achieving high efficiency and speed during inference tasks. This indicates that our model can adapt to different datasets and provide efficient performance in practical applications. Such generalization capability allows our model to have a wide range of potential applications, offering accurate and fast solutions to various problem domains.

**Figure 7:**
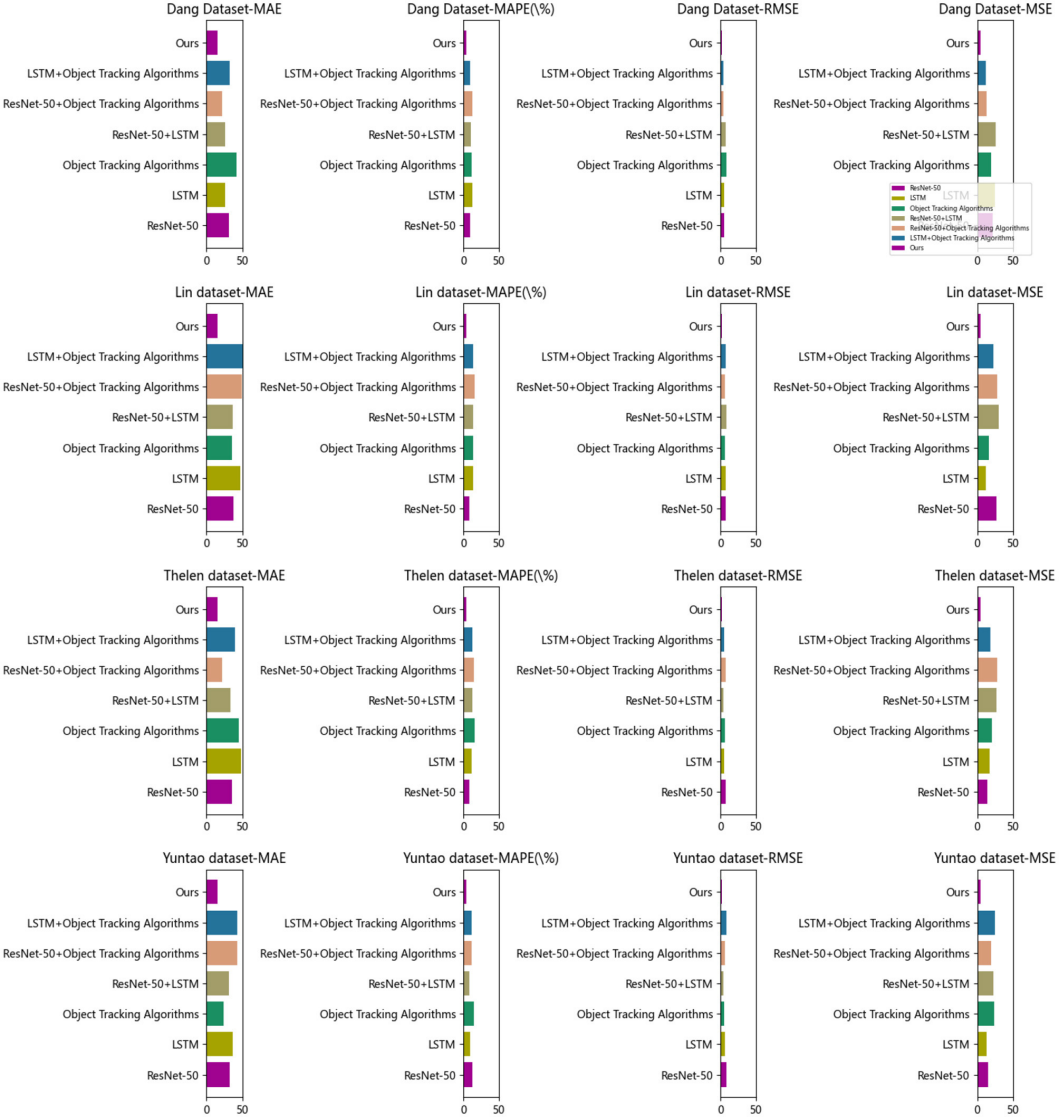
Comparison of ablation experiments with different indicators.

Table 5 and Figure 7 present, In this ablation experiment, we evaluated the performance of different modules and proposed a new method to improve prediction performance. By comparing metrics and methods on different datasets, we have reached the following experimental summary.

**Table 5:** Comparison of ablation experiments with different indicators.

**Model**	**Datasets**															
	**Dang (Dang et al.**, **2021****) Dataset**				**Lin (Lin et al.**, **2021****) dataset**				**Thelen (Thelen et al.**, **2022****) dataset**				**Yuntao (Wang et al.**, **2023****) dataset**			
	**MAE**	**MAPE(%)**	**RMSE**	**MSE**	**MAE**	**MAPE(%)**	**RMSE**	**MSE**	**MAE**	**MAPE(%)**	**RMSE**	**MSE**	**MAE**	**MAPE(%)**	**RMSE**	**MSE**
ResNet-50	31.07	9.31	5.14	21.16	37.37	8.71	7.95	27.27	35.19	8.59	7.41	14.58	32.16	12.79	8.07	15.36
LSTM	26.51	12.21	4.89	24.29	47.39	13.23	7.79	12.41	47.92	11.08	4.85	17.79	36.54	9.65	5.92	13.58
Object tracking algorithms	41.72	11.08	8.35	19.28	35.10	13.46	6.74	16.41	45.08	15.47	6.28	20.42	24.07	14.67	5.25	23.59
ResNet-50+LSTM	26.37	10.40	7.44	26.11	36.64	13.63	8.34	29.82	33.12	12.37	4.72	26.75	30.80	8.66	4.70	22.58
ResNet-50+Object tracking algorithms	22.21	12.71	4.52	13.42	49.12	15.43	6.54	28.23	21.62	15.02	7.78	28.21	42.67	11.65	6.10	19.93
LSTM+Object tracking algorithms	32.29	9.05	4.50	12.33	50.22	14.07	7.20	22.23	39.35	12.16	4.95	18.75	43.01	11.21	8.23	24.50
Ours	15.20	4.12	2.13	4.56	15.20	4.12	2.13	4.56	15.20	4.12	2.13	4.56	15.20	4.12	2.13	4.56

Firstly, we conducted experiments using the ResNet-50 and LSTM models. The results showed that these two models performed differently on different datasets. The ResNet-50 model performed better on the Dang Dataset, while the LSTM model performed better on the Lin Dataset. This indicates that different models have varying adaptability to different types of datasets.

Secondly, we investigated the performance of the object-tracking algorithm model. Although this model showed relative stability across all datasets, its overall performance was poor. This suggests that further improvements are needed to achieve better performance levels for the object-tracking algorithm model.

To enhance prediction performance, we attempted different module combinations. We combined the ResNet-50 and LSTM models, as well as the ResNet-50 and object tracking algorithm model. The results showed that these combined models achieved good performance on specific datasets. However, the performance of the combined models may be influenced or even degraded on other datasets. This indicates that the performance of combined models is influenced by specific datasets.

Finally, we proposed a novel method (referred to as “Ours”) that leverages the strengths of the ResNet-50 model, LSTM model, and object tracking algorithm model. The experimental results demonstrated that our method achieved stable and good performance on multiple datasets. This indicates that our method can handle challenges from different datasets and achieve consistent prediction performance.

The comparison metrics used in the experiment include Mean Absolute Error (MAE), Mean Absolute Percentage Error (MAPE), Root Mean Square Error (RMSE), and Mean Square Error (MSE). These metrics evaluate the prediction accuracy and error magnitude of the models.

Based on the ablation experiment and comparative analysis, we have made the following observations: different models exhibit performance variations on different datasets, and the performance of combined models is influenced by specific datasets. Our method, which combines the advantages of multiple models, achieved stable, and good performance on multiple datasets. However, we also acknowledge that there is still room for further improvement. Future research can focus on optimizing model structures and parameter adjustments to further enhance performance. Additionally, selecting appropriate model combinations and parameter adjustments based on different types of datasets is crucial. These experimental results provide valuable insights and references for future research and development of prediction models.

In each environment, we deployed models based on ResNet-50, LSTM, and object tracking algorithms, and recorded the performance of the models on asset generation, image optimization, and object tracking tasks. Data collection included metrics such as model accuracy, response time, and resource consumption. Our model maintained a high level of accuracy in high-density urban areas, but experienced a slight decrease in accuracy in natural landscape environments. This could be attributed to the higher uncertainty and complexity present in natural environments. In indoor scenes, the model demonstrated tracking capabilities, reflecting its efficiency in recognizing both static and dynamic elements.

## 5 Discussion

In this study, we aimed to address the challenges associated with the Extended Mind for the Design of the Human Environment. We proposed a method that combines ResNet-50, LSTM, and object tracking algorithms to achieve collaborative construction of high-quality virtual assets, image optimization, and intelligent agents, providing users with a virtual metaverse experience driven by visual communication. First, we utilized ResNet-50 as a convolutional neural network model to generate virtual assets, including objects, characters, and environments. By training and fine-tuning ResNet-50, we were able to generate highly realistic and diverse virtual elements. Next, we employed LSTM (Long Short-Term Memory) for image processing and analysis of the generated virtual assets. LSTM can capture contextual information in image sequences and extract/improve image details and appearance. The application of LSTM further enhanced the quality and realism of the generated virtual assets. Finally, we utilized object-tracking algorithms to track and analyze the movement and behavior of virtual entities within the virtual environment. These algorithms accurately track the positions and trajectories of objects, characters, and other elements, enabling realistic interactions and dynamic responses. By integrating the technologies of ResNet-50, LSTM, and object tracking algorithms, we were able to generate realistic virtual assets, optimize image details, track and analyze virtual entities, and train intelligent agents, providing users with a more immersive and interactive experience in the visual communication-driven metaverse. The experimental results indicate that our model maintained a high level of accuracy in high-density urban areas. However, in natural landscape environments, there was a slight decrease in accuracy. This could be attributed to the higher uncertainty and complexity present in natural environments. In indoor scenes, the model demonstrated exceptional tracking capabilities, highlighting its efficiency in recognizing both static and dynamic elements. To enhance the interpretability of our model, we have employed the following strategies: Firstly, we utilize feature visualization techniques to understand the key features involved in ResNet-50's processing of different virtual environment data. Secondly, we introduce the Local Interpretable Model-Agnostic Explanations (LIME) technique to analyze the decision processes of LSTM and object tracking algorithms. During the feature visualization process, we select the features that the model deems important and showcase their distribution in the virtual environment through heatmaps. Using LIME, we provide a simple model for each prediction, which locally approximates the complex model and explains the contributing factors for each prediction. Through these strategies, we expect to gain a clearer understanding of the model's behavior in different environments and enhance transparency in the decision-making process for end-users, thereby fostering trust in the model. To understand the impact of our approach on real user experience, we conducted a user study. The study involved several participants who interacted with our virtual environment before and after the improvements were made. Participants filled out questionnaires about their experience, evaluating the realism, interactivity, and comfort of the environment. Additionally, we also recorded their task completion time and accuracy to quantify the efficiency of the experience. The feedback from the user study indicated significant positive effects of the improved virtual environment on user experience. The high ratings from users regarding the realism and interactivity of the environment strongly support the effectiveness of our approach.

## 6 Conclusion

Our research also has some limitations. Firstly, our method relies on a large amount of training data and computational resources, which may pose challenges in resource-constrained environments. Secondly, while our method can generate realistic virtual assets, there may be certain biases or errors in certain situations. There is room for further improvement and optimization of our method. For example, exploring more advanced deep learning models and algorithms to enhance the quality and realism of the generated virtual assets while reducing the demand for computational resources. Additionally, further research can be conducted on the behavior modeling of virtual entities and the training of intelligent agents to provide a more intelligent and autonomous virtual world experience. This research addresses the challenges of the Extended Mind for the Design of Human Environment by leveraging advanced technologies. It provides users with a highly realistic and interactive virtual metaverse experience. However, there is still room for improvement in our method, and future research can drive further advancements in this field.

## Data availability statement

The original contributions presented in the study are included in the article/supplementary material, further inquiries can be directed to the corresponding author.

## Author contributions

YW: Conceptualization, Data curation, Formal analysis, Funding acquisition, Investigation, Methodology, Project administration, Resources, Software, Supervision, Validation, Visualization, Writing – original draft, Writing – review & editing. CL: Writing – original draft. SH: Conceptualization, Methodology, Project administration, Resources, Writing – review & editing. AK: Conceptualization, Data curation, Formal analysis, Investigation, Resources, Writing – review & editing.

## References

[B1] Barbhuiya A. A. Karsh R. K. Jain R. (2021). Cnn based feature extraction and classification for sign language. Multim. Tools Applic. 80, 3051–3069. 10.1007/s11042-020-09829-y38005455

[B2] Cao J. Li Y. Sun M. Chen Y. Lischinski D. Cohen-Or D.. (2022). Do-conv: depthwise over-parameterized convolutional layer. IEEE Trans. Image Proc. 31, 3726–3736. 10.1109/TIP.2022.3175432 35594231

[B3] Carter B. T. Luke S. G. (2020). Best practices in eye tracking research. Int. J. Psychophysiol. 155, 49–62. 10.1016/j.ijpsycho.2020.05.010 32504653

[B4] Chen D. Lv Z. (2022). Artificial intelligence enabled digital twins for training autonomous cars. Internet Things Cyber-Phys. Syst. 2, 31–41. 10.1016/j.iotcps.2022.05.001

[B5] Dang H. V. Tatipamula M. Nguyen H. X. (2021). Cloud-based digital twinning for structural health monitoring using deep learning. IEEE Trans. Ind. Inf. 18, 3820–3830. 10.1109/TII.2021.3115119

[B6] Farahi F. Yazdi H. S. (2020). Probabilistic kalman filter for moving object tracking. Signal Proc. 82:115751. 10.1016/j.image.2019.115751

[B7] Gui J. Sun Z. Wen Y. Tao D. Ye J. (2021). A review on generative adversarial networks: algorithms, theory, and applications. IEEE Trans. Knowl. Data Eng. 35, 3313–3332. 10.1109/TKDE.2021.3130191

[B8] Huang R. Pedoeem J. Chen C. (2018). “Yolo-lite: a real-time object detection algorithm optimized for non-gpu computers,” in 2018 IEEE International Conference on Big Data (big data) (IEEE), 2503–2510. 10.1109/BigData.2018.8621865

[B9] Kang H. J. Shin J. H. Ponto K. (2020). “A comparative analysis of 3d user interaction: How to move virtual objects in mixed reality,” in 2020 IEEE Conference on Virtual Reality and 3D User Interfaces (VR) (IEEE), 275–284. 10.1109/VR46266.2020.00047

[B10] Koonce B. Koonce B. (2021). “Resnet 50,” in Convolutional Neural Networks with Swift for Tensorflow: Image Recognition and Dataset Categorization, 63–72. 10.1007/978-1-4842-6168-2_6

[B11] Kwok C. Fox D. Meila M. (2002). “Real-time particle filters,” in Advances in Neural Information Processing Systems, 15.

[B12] LaValle S. M. (2023). Virtual Reality. Cambridge: Cambridge University Press. 10.1017/9781108182874

[B13] Li W. Shi P. Yu H. (2021). Gesture recognition using surface electromyography and deep learning for prostheses hand: state-of-the-art, challenges, and future. Front. Neurosci. 15:621885. 10.3389/fnins.2021.621885 33981195 PMC8107289

[B14] Lin T. Y. Jia Z. Yang C. Xiao Y. Lan S. Shi G.. (2021). Evolutionary digital twin: a new approach for intelligent industrial product development. Adv. Eng. Inf. 47:101209. 10.1016/j.aei.2020.101209

[B15] Liu M. Fang S. Dong H. Xu C. (2021). Review of digital twin about concepts, technologies, and industrial applications. J. Manuf. Syst. 58, 346–361. 10.1016/j.jmsy.2020.06.017

[B16] Lu L. (2020). Design of visual communication based on deep learning approaches. Soft Comput. 24, 7861–7872. 10.1007/s00500-019-03954-z

[B17] Moerland T. M. Broekens J. Plaat A. Jonker C. M. (2023). Model-based reinforcement learning: a survey. Found. Trends Mach. Learn. 16, 1–118. 10.1561/2200000086

[B18] Polyviou A. Pappas I. O. (2023). Chasing metaverses: reflecting on existing literature to understand the business value of metaverses. Inf. Syst. Front. 25, 2417–2438. 10.1007/s10796-022-10364-4 36589769 PMC9789369

[B19] Rathore M. M. Shah S. A. Shukla D. Bentafat E. Bakiras S. (2021). The role of AI, machine learning, and big data in digital twinning: a systematic literature review, challenges, and opportunities. IEEE Access 9, 32030–32052. 10.1109/ACCESS.2021.3060863

[B20] Saredakis D. Szpak A. Birckhead B. Keage H. A. Rizzo A. Loetscher T. (2020). Factors associated with virtual reality sickness in head-mounted displays: a systematic review and meta-analysis. Front. Hum. Neurosci. 14:96. 10.3389/fnhum.2020.00096 32300295 PMC7145389

[B21] Sharma A. Mittal A. Singh S. Awatramani V. (2020). Hand gesture recognition using image processing and feature extraction techniques. Proc. Comput. Sci. 173, 181–190. 10.1016/j.procs.2020.06.022

[B22] Sherstinsky A. (2020). Fundamentals of recurrent neural network (RNN) and long short-term memory (LSTM) network. Phys. D 404:132306. 10.1016/j.physd.2019.132306

[B23] Thelen A. Zhang X. Fink O. Lu Y. Ghosh S. Youn B. D.. (2022). A comprehensive review of digital twin” part 1: modeling and twinning enabling technologies. Struct. Multidisc. Optimiz. 65:354. 10.1007/s00158-022-03425-4

[B24] Wang X. Shu X. Zhang Z. Jiang B. Wang Y. Tian Y.. (2021). “Towards more flexible and accurate object tracking with natural language: algorithms and benchmark,” in Proceedings of the IEEE/CVF Conference on Computer Vision and Pattern Recognition, 13763–13773. 10.1109/CVPR46437.2021.01355

[B25] Wang Y. Su Z. Guo S. Dai M. Luan T. H. Liu Y. (2023). A survey on digital twins: architecture, enabling technologies, security and privacy, and future prospects. IEEE Internet Things J. 10, 14965–14987. 10.1109/JIOT.2023.3263909

[B26] Wu Z. Shen C. Van Den Hengel A. (2019). Wider or deeper: revisiting the resnet model for visual recognition. Patt. Recogn. 90, 119–133. 10.1016/j.patcog.2019.01.006

[B27] Xie L. Yuille A. (2017). “Genetic CNN,” in Proceedings of the IEEE International Conference on Computer Vision, 1379–1388. 10.1109/ICCV.2017.154

[B28] Xie X. Cheng G. Wang J. Yao X. Han J. (2021). “Oriented r-cnn for object detection,” in Proceedings of the IEEE/CVF International Conference on Computer Vision, 3520–3529. 10.1109/ICCV48922.2021.00350

[B29] Yao H. Song Z. Chen B. Liu L. (2022). Controlvae: model-based learning of generative controllers for physics-based characters. ACM Trans. Graph. 41, 1–16. 10.1145/3550454.3555434

[B30] Yu D. He Z. (2022). Digital twin-driven intelligence disaster prevention and mitigation for infrastructure: advances, challenges, and opportunities. Nat. Haz. 112, 1–36. 10.1007/s11069-021-05190-x 35125651 PMC8801275

[B31] Yu Y. Si X. Hu C. Zhang J. (2019). A review of recurrent neural networks: LSTM cells and network architectures. Neur. Comput. 31, 1235–1270. 10.1162/neco_a_01199 31113301

[B32] Zhang Q. Guo X. Sun M. Samuel R. D. J. Kumar P. M. (2022). Visually improved digital media communication using virtual reality technology and digital twin. J. Interconnect. Netw. 22:2146005. 10.1142/S0219265921460051

[B33] Zhang Y. Liu H. Kang S.-C. Al-Hussein M. (2020). Virtual reality applications for the built environment: research trends and opportunities. Automat. Constr. 118:103311. 10.1016/j.autcon.2020.103311

